# Reduced Duration of Postchikungunya Musculoskeletal Pain in Rheumatological Patients Treated with Biologicals

**DOI:** 10.1155/2020/2071325

**Published:** 2020-07-10

**Authors:** Carlos A. A. de Brito, Claudia D. L. Marques, Rafael F. O. França, Janaína R. Monteiro, Marina C. M. de Brito, Fabiana Lopes, Anton Lima, Gabriel L. Marques, Maria Luisa Valadares, Angela L. B. P. Duarte

**Affiliations:** ^1^Internal Medicine, Clinical Hospital of Federal University of Pernambuco and Autoimmune Institute, Recife, Pernambuco, Brazil; ^2^Rheumatology Service, Clinical Hospital of Federal University of Pernambuco, Recife, Pernambuco, Brazil; ^3^Department of Virology and Experimental Therapy, Aggeu Magalhães Institute, Oswaldo Cruz Foundation (FIOCRUZ), Recife, Pernambuco, Brazil; ^4^Federal University of Pernambuco, Recife, Pernambuco, Brazil; ^5^Universidade Católica de Pernambuco (UNICAP), Recife, Pernambuco, Brazil; ^6^Faculdade de Medicina de Olinda (FMO), Olinda, Pernambuco, Brazil

## Abstract

Chikungunya fever (CHIK) has caused important epidemic outbreaks in the Americas, with musculoskeletal involvement being the main manifestation, causing chronic symptoms in half of the affected patients. This study was performed to evaluate the clinical course of the infection in 168 patients with autoimmune inflammatory disease using biological disease-modifying antirheumatic drugs (bDMARDs), comparing this group with 56 household controls. Anti-CHIKV IgG serology was positive in 42 (25%) of the patients in the bDMARD group and in 15 (27%) of the controls (*p*=0.79). Of those with positive serology, 32 (76%) and 14 (93%) were symptomatic among subjects in the bDMARD and control groups, respectively (*p*=0.87). Persistence of musculoskeletal symptoms for more than three months occurred in 64% of the patients in the control group and only in 28% in the bDMARD group (*p*=0.021), maintaining a statistically significant difference only for users of anti-TNF. This study found that patients affected by chikungunya fever using bDMARDs did not present severe forms or complications in the acute phase of the disease, and patients using anti-TNF biologicals had a lower frequency of chronic joint symptoms than the household controls. This favorable outcome may be related to the cytokine blockade, with a reduction in the inflammatory response and joint damage.

## 1. Introduction

Chikungunya fever (CHIK) is a mosquito-borne disease, caused by an alphavirus responsible for large outbreaks, with reports of over 10 million cases in a decade worldwide. In the Americas, 2.7 million cases have been reported between the discovery of the virus in 2013 and 2017, with 773 thousand (29%) occurring in Brazil, mostly in Northeast Brazil in the state of Pernambuco, which was the epicenter of an epidemic that occurred in 2017 [1, 2]. Clinically, the disease is characterized by fever and joint pain that is generally disabling in the acute phase and may last from months to years, with the potential to evolve into chronic musculoskeletal symptoms in approximately 30–40% of cases [3]. In addition, the spectrum of the disease includes severe and atypical forms, with involvement of the central and peripheral nervous system, heart, kidneys, lungs, liver, and circulatory system [4–7].

In this scenario of reemergence of outbreaks, there is a concern about the intensity of the disease in the acute phase, which may cause incapacity, and the risk of complications in users of immunosuppressive drugs, especially patients with rheumatological disease who already have joint involvement [8, 9].

The use of immunosuppressive drugs such as biological disease-modifying antirheumatic drugs (bDMARDs) is often applied in rheumatological diseases. Despite the benefit of the use of bDMARDs, these drugs are known to have immunomodulatory and immunosuppressive effects, predisposing the users to an increased risk of infections and their complications by viruses such as cytomegalovirus (CMV), varicella zoster virus, Epstein-Barr virus, hepatitis B virus, and influenza virus [8–10]. On the other hand, bDMARDs have the potential to block inflammatory cytokines, can reduce the inflammatory process in tissues induced by viral infection, and may have benefits in the outcome of some diseases that have a chronic pattern, such as chikungunya fever [11–13].

Thus, based on the above information and the possibility of emergence of new epidemics caused by CHIKV, it is necessary to assess the disease pattern in users of these immunosuppressive drugs, defining the risks and possible benefits and contributing to future therapeutic guidelines for this group of patients.

The aim of this study was to describe the clinical pattern and evolution of CHIK in patients with chronic inflammatory arthropathy using bDMARDs and to verify the frequency of asymptomatic CHIKV infection compared with that of healthy household contacts.

## 2. Materials and Methods

### 2.1. Design, Location, and Period of Study

We conducted a retrospective cross-sectional study that included patients diagnosed with chronic inflammatory rheumatologic disease using bDMARDs who were treated at the Rheumatology Service of the Clínicas' Hospital of the Federal University of Pernambuco (UFPE) and at the Biological Therapy Unit of Jayme da Fonte Hospital (Recife, PE) from October 2016 to April 2017.

### 2.2. Participants

The cases were patients older than 18 years who were diagnosed with chronic inflammatory arthropathy (rheumatoid arthritis, ankylosing spondylitis, or psoriatic arthritis) and who used bDMARD.

For the comparison group (control), we selected people with no diagnosis of inflammatory disease or use of immunosuppressants who lived in the same house. The choice for this group aimed to include a control that was in the same environment and with potentially the same risk of exposure to the vector *Aedes aegypti*.

Only patients who did not provide consent to participate in the study were excluded.

### 2.3. Data Collection

Patients were recruited on the day of routine outpatient care and invited to participate in the study. If the patient was accompanied by a person residing in the same household, he or she was also invited to participate in the study. After reading and signing the free and informed consent, a standardized questionnaire was applied by a previously trained researcher to cases and controls to collect clinical information about suggestive symptoms of CHIKV infection between December 2016 and May 2017, the period of the epidemic in our state. The questionnaires were applied in a period of three to six months subsequent to the epidemic period. Patients from endemic or epidemic areas with a history of sudden onset fever >38.5°C and severe acute onset arthralgia or arthritis not explained by other conditions were considered to be suspected cases (epidemiological clinical criteria).

Information was collected about the rheumatologic disease, class of bDMARD in use, duration of symptoms suggestive of CHIK after acute infection, comorbidities, smoking history, intensity of pain in the acute and chronic phases by visual analog scale (VAS) from 0 to 10, pattern of joint involvement (mono-, oligo-, or polyarticular), and possible complications or atypical manifestations in the acute phase.

Patients who met the clinical criteria of suspected cases of acute CHIK with positive IgG serology and a history of persistent musculoskeletal symptoms of the acute phase for a period longer than three months were considered as cases with chronic involvement.

Regarding the clinical pattern of musculoskeletal pain, the cases in which there was no period of symptom improvement or arthralgia remission after the acute episode of chikungunya until the interview or complete regression of the condition were considered to exhibit the “persistent” pattern. In the “intermittent” or “relapsing” pattern, there were periods of improvement or remission, followed by exacerbations with the same characteristics as those of the acute phase, between the period from the onset of the condition until the interview or complete regression.

Five milliliters of blood was collected and analyzed at FIOCRUZ/PE following the standard operating procedure (SOP). We used an ELISA (EUROIMMUN, Lübeck, Schleswig-Holstein, Germany) to identify IgG antibodies specific to CHIKV according to the manufacturer's instructions [14].

### 2.4. Ethical Aspects

This study complies with the Declaration of Helsinki and was evaluated and approved by the Research Ethics Committee of the UFPE Health Science Center, CAAE 54275516.9.1001.5208, approved on July 12, 2016, under number 1.632.342. Informed consent was obtained from all the subjects (or their legally authorized representatives).

### 2.5. Statistical Analysis

Descriptive statistics were performed for numerical variables through the range and standard deviation (SD) or median and interquartile range (IQR). When the distribution was considered nonnormal, the categorical variables were described as numbers and percentages. The chi-square test was used to verify the differences between the proportions, and Student's *t*-test was used to evaluate the differences between the means. The confidence interval for proportions was calculated, as well as the absolute risk reduction (AAR), to compare the results between symptomatic patients with and without bDMARD. The confidence interval was 95%. Data were analyzed using Stata Statistical Software, version 16.

## 3. Results

### 3.1. Demographic Data, Comorbidities, Rheumatological Disease, and bDMARD Use

We evaluated 168 patients using bDMARDs and 56 household controls with a mean age of 50.15 years (±13.92) and 48.47 (±16.85), respectively (*p*=0.314). Female sex corresponded to 67% of the cases in the group using bDMARDs, which was more frequent than in the controls, with 52% females, but the difference was not statistically significant (*p*=0.055). The presence of comorbidities was observed in 80% of the group of patients using bDMARDs and in 57% of the control group (*p*=0.001).

Regarding the group having chronic inflammatory arthropathy and using bDMARDs, 94 (56%) subjects had a diagnosis of rheumatoid arthritis, and the most frequent class of immunobiological used was antitumor necrosis factor biologicals (69%) (Table 1).

### 3.2. Symptoms of CHIKV and Seropositivity

Suggestive symptoms of CHIKV infection were reported by 51/168 (30%) of the patients in the group using bDMARDs and in 21/56 (37.5%) of the control group subjects (*p*=0.397), but serology for anti-CHIKV IgG was positive in only 42/168 (25%) and in 15/56 (27%) for the cases and controls, respectively, without a statistically significant difference between the groups (*p*=0.79).

Of the 42 seropositive individuals in the bDMARD user group, 32 (76%) were symptomatic, and among the controls, 14 (93%) of the 15 seropositive individuals developed symptoms (*p*=0.87). The demographic clinical data of the sample can be seen in Table 1.

### 3.3. Clinical Manifestations and Intensity of Arthralgia

Regarding symptoms suggestive of CHIK among patients with positive anti-CHIKV IgG, the most frequent was joint pain (98.6%), followed by fever (82%), arthritis (75%), and myalgia (75%).

No significant difference was observed in the frequency of symptoms between the two groups. There was no difference in acute pain intensity between the two groups (VAS 8.78 in the bDMARD group; 9.04 in the control group; *p*=0.615) or in the chronic phase (VAS 5.23 in the bDMARD group; 5.08 in the control group; *p*=0.85). No cases of complications or death were reported in either group.

### 3.4. Duration of Symptoms

Of the 32 patients with symptoms and anti-CHIKV IgG positivity in the bDMARD group, 28% had symptoms lasting more than three months, while in the control group, this presentation occurred in 64.2% of subjects (*p*=0.021). When analyzed by bDMARD class and compared with the control group, only the patients using anti-TNF biologicals showed statically significant difference (*p* < 0.001) (Table 2).

## 4. Discussion

In our study, we observed that the use of bDMARDs was not associated with severe, atypical forms and complications of chikungunya fever, while it was associated with a shorter duration of symptoms, particularly with the use of anti-TNF biologicals, and a larger number of asymptomatic infection cases, although without statistical significance.

A few published studies have analyzed the risks of rheumatologic patients using bDMARDs and CHIKV-infected patients, and published results are similar to those in our study, although with different methodologies [15, 16].

Experimental models of alphavirus-induced arthritis suggest that CHIK pathogenesis is the result of a combination of direct cellular and tissue damage caused by viral replication and mainly by activation of the indirect immune response in target tissues, with the production of different cytokines, such as interleukins and TNF [17–19]. Venugopalan et al. demonstrated a correlation between elevated TNF levels and the presence of symptoms at different stages of the disease [19]. Since TNF is one of the elements involved in the inflammatory response and tissue damage, its blockade or reduced synthesis could potentially slow the inflammatory process in tissues in the acute phase of CHIKV infection. Our finding of this more favorable clinical evolution in our patients using anti-TNF biologicals than that in controls is probably due to the reduction in the intensity of inflammation by blocking cytokines involved in the pathophysiology of chronic joint injury.

The seroprevalence of anti-CHIKV IgG observed in our study was similar for the group of patients using bDMARDs and the control group, reaching approximately 25% of the patients, corroborating the results of the study by Cunha et al., who found a seroprevalence of 20.0% in Feira de Santana, northeastern Brazil [20]. Although no statistically significant difference was demonstrated, the number of subjects in the bDMARD group with positive serology and no symptoms was higher than that in the control group, suggesting that despite immunosuppression, there was no increased risk of symptomatic infections.

Another relevant finding was that most symptomatic bDMARD-using patients had a shorter CHIK-related symptom duration than controls, with only 28% suffering symptoms beyond three months. When analyzing patients separately by immunobiological class, this positive result was only statistically significant in the group using anti-TNF drugs. The percentage of chronicity presented by controls was similar to the literature data, with approximately 50% of patients persisting with joint complaints for more than three months [3, 21].

A previous study using mice infected with an arbovirus (Ross River virus), in which etanercept was applied in the acute phase, showed a higher viral load and higher recruitment of inflammatory cells and tissue damage than mice without etanercept, leading to the death of all tested animals using the drug on the 14th day and suggesting that the use of anti-TNF biologicals could aggravate the disease [22]. However, this result is not reproduced in clinical practice, although the use of anti-TNF biologicals in the acute phase has not been evaluated exclusively. Two studies in the literature refer to the pattern of CHIKV infection in biological users and do not show a more severe joint disease in this group of patients [15, 16].

Rosario V. et al. described 53 cases of patients with rheumatoid arthritis (RA) using different immunobiologicals either alone or associated with other disease-modifying drugs (methotrexate, leflunomide, sulfasalazine, and hydroxychloroquine), with polyarthralgia and arthritis occurring in 96.2% and 47% of the cases, without differences when compared to a group of patients with chikungunya without a history of previous arthropathy; however, the duration of musculoskeletal symptoms was not evaluated and compared [15]. In the other series of cases, clinical manifestations were reported in 22 patients using biologicals (16 patients using anti-TNF biologicals and abatacept or tocilizumab in six other cases), suggesting that the articular manifestations in users of biologicals would not be more intense than those described in the literature, but there was no comparison group in the study [16].

This beneficial effect of cytokine blockade has been reported in other viral infections, such as dengue, Zika, influenza, and, most recently, in coronavirus infections due to the pandemic of COVID-19 [11–13, 23–25]. An animal model study with the BALB/c mouse strain infected with dengue virus type 2 identified elevated levels of TNF-*α* within the first 24 hours, with a 60% reduction in lethality in mice treated with anti-TNF [11]. The administration of infliximab, a TNF-alpha blocker, in mice infected with Zika virus was able to attenuate lesions of the central nervous [13].

Although a favorable outcome has been shown in this study, the current recommendation of the Brazilian Society of Rheumatology for patients using bDMARDs with clinical-epidemiological or laboratory diagnosis of arbovirus infection is to suspend biological therapy until recovery from the acute phase of the disease [21].

The limitations of this study include the retrospective cross-sectional character, leading to possible memory biases; however, as the inclusion of the patients in this study occurred immediately after the period of the epidemic and the symptoms of chikungunya are intense and characteristic, this tendency is likely to decrease. It was also not possible to include a matched number of cases in the control group since most patients came to the consultation unaccompanied, and it was not possible to form a larger sample of controls due to the fact that the socioeconomical conditions of those patients were low and it was not possible for more than one person of the household to go to the appointments to answer the questionnaires. Perhaps with a larger sample, it would be possible to demonstrate the significant difference in the number of cases with subclinical infection between the two groups.

## 5. Conclusion

In patients with chronic inflammatory arthropathy, the use of bDMARDs does not seem to be associated with worse outcomes in patients infected by CHIKV, and we observed a lower frequency of chronic joint symptoms that may be related to possible TNF blockade in the presence of CHIKV infection.

In this study, patients with chronic inflammatory arthropathy using bDMARDs did not present severe forms of CHIK or complications related to the acute disease. A lower frequency of chronicity of joint symptoms was observed, compared to that described in the literature and to healthy non-bDMARD-using family members of this case series.

One hypothesis is that the mechanisms of action of many of these drugs do not significantly interfere with the immune response mechanisms against these viral infections, facilitate viral replication, or play a role in the worsening of the disease. On the other hand, these drugs may reduce the intensity of inflammation by blocking cytokines, such as TNF, involved in the pathophysiology of severe forms of arbovirus infections.

Further studies are needed to better assess the behavior of this disease in biological therapy users, helping define the risks and benefits and guide the therapeutic approach to chikungunya infection.

## Figures and Tables

**Table 1:** Clinical and demographic data comparing the group with rheumatologic disease using bDMARDs and the control group.

		bDMARD (*N* = 168)	Control (*N* = 56)	*p* value
Age (mean. ± SD)^#^		50.68 (12.86)	48.47 (16.85)	0.314
Sex—*N* (%)				
Female		113 (67)	29 (52)	0.055
Male		55 (33)	27 (48)	
Comorbidities—*N* (%)		134 (80)	32 (57)	0.001
Hypertension		65 (39)	14 (25)	0.231
Diabetes		24 (14)	9 (16)	0.359
Psoriasis		18 (11)	0 (0)	0.026
Respiratory disease		20 (12)	3 (5)	0.405
Smoking		6 (4)	7 (13)	0.002
Cardiovascular disease		11 (6.5)	5 (9)	0.341
Renal disease		4 (2)	2 (4)	0.610
Inflammatory bowel disease		7 (4)	0 (0)	0.190
Rheumatologic diagnosis—*N* (%)				
Rheumatoid arthritis		94 (56)	—	—
Ankylosing spondylitis		44 (26)	—	—
Psoriatic arthritis		18 (11)	—	—
Spondyloarthritis		8 (5)	—	—
Psoriasis		2 (1)	—	—
Uveitis/Behçet disease		2 (1)	—	—
bDMARD—*N* (%)		168 (100)	—	—
Anti-TNFc^*∗*^		116 (69)	—	—
Others^*∗∗*^		52 (31)	—	—
sDMARD—*N* (%)		71 (42)	—	—
Methotrexate		49 (29)	—	—
Leflunomide		17 (10)	—	—
Sulfasalazine		5 (3)	—	—
Prednisone		49 (29)	—	—
CHIK symptoms		51 (30)	21 (37)	0.397
Musculoskeletal symptoms				
<3 months		36 (21)	12 (21)	0.222
>3 months		15 (9)	9 (16)	
Pain pattern				
Persistent		21 (13)	8 (14)	0.835
Polyarticular		38 (22)	14 (25)	0.510
Anti-CHIKV IgG				
Positive		42 (25)	15 (27)	0.79
Negative	126 (75)	41 (73)		

TNF: tumor necrosis factor; bDMARD: biological disease-modifying antirheumatic drug. ^#^*t*-test; ^*∗*^infliximab, etanercept, adalimumab, golimumab, or certolizumab; ^*∗∗*^abatacept, rituximab, tocilizumab, or secukinumab.

**Table 2:** Duration of CHIK symptoms comparing patients with positive IgG using biological DMARDs with the controls and comparing anti-TNF biologicals with other bDMARDs.

Symptom duration	bDMARD *N* (%)	Control *N* (%)	Total	*p* value (ARR; 95% CI)

<3 months	23 (72%)	5 (36%)	28	0.021 (−0.362; −0.457 to −0.266)
>3 months	9 (28%)	9 (64%)	18	

	*Anti-TNF*	*Control*		

<3 months	19 (95%)	5 (36%)	24	0.001 (−0.593; −0.748 to −0.437)
>3 months	1 (5%)	9 (64%)	10	

	*Other bDMARD*	*Control*		

<3 months	4 (33%)	5 (36%)	9	0.899 (0.024; 0.040 to 0.008)
>3 months	8 (67%)	9 (64%)	17	

bDMARD: biological disease-modifying antirheumatic drug; ARR: absolute risk reduction; other bDMARD included abatacept, rituximab, tocilizumab, and secukinumab.

## Data Availability

The data used to support the findings of this study are included within the article.

## Additional Points

*Keypoints* . bDMARDs are not associated with worsened outcomes in patients with CHIKV infection. Anti-TNF biological use may reduce symptom duration in chikungunya fever patients.
